# Asymmetrical flow field-flow fractionation and multi-angle laser light scattering: A new analytical approach for the characterisation of insect protein aggregation/polymerisation after heat treatment of *Tenebrio molitor* larvae

**DOI:** 10.1016/j.crfs.2025.101077

**Published:** 2025-05-17

**Authors:** Ariel Anouma, Céline Niquet-Léridon, Bénédicte Lorrette, Thierry Aussenac

**Affiliations:** a Institut Polytechnique UniLaSalle, Université d’Artois, ULR 7519, 19 rue Pierre Waguet, BP 30313, Beauvais, 60026, France; b YNSECT, 1 rue Pierre Fontaine, Evry, 91000, France

**Keywords:** *Tenebrio molitor*, Proteins, Physicochemical properties, AF4-MALLS characterisation, Structural modifications

## Abstract

Understanding the structural modifications of insect proteins during the transformation processes used for extract preparation is essential for optimising their functionalities and obtaining high added-value proteins. From this perspective and in addition to classical analytical approaches, we developed an original methodology based on the implementation of Asymmetrical Flow Field-Flow Fractionation and Multi-Angle Laser Light Scattering (A4F-MALLS) coupling to quantify and characterise the aggregation/polymerisation phenomena of *Tenebrio molitor* larvae proteins after heat treatment (from 65 to 95 °C). Applied to heat-treated larvae proteins in conjunction with the evaluation of intrinsic fluorescence, surface hydrophobicity and sodium dodecyl sulphate polyacrylamide gel electrophoresis (SDS-PAGE), the AF4-MALLS method enabled us to quantify and characterise the aggregated proteins (forms dissociable after urea denaturation), determining the polymer/monomer (P/M) ratio. Heat treatment significantly affects solubility (−35 %), which is due to the amplification of aggregation phenomena, as demonstrated by the increase in the P/M ratio ( × 10). Moreover, the method enabled us to quantify and characterise the polymerised protein (forms dissociable after chemical reduction of intermolecular bonds), identifying the elements by molar mass and size distribution and conformation. Proteins with cysteine groups can be polymerised under heat, causing a thiol-disulphide exchange reaction and forming a strong (M_w_ > 10^7^ g/mol, R_Gw_ >130 nm) and compact polymer structure (*v* ≤ 0.35) and resulting in intermolecular S-S bonds that preferentially mobilise proteins with M_w_ > 80 kDa. Given its performances, the AF4-MALLS method is a real opportunity to understand the effects of processing methods, such as thermal and non-thermal treatments, to optimise protein functionalities.

## Highlights

• Heat treatment of larvae proteins significantly promote their aggregation.
• A4F-MALLS coupling allow the quantification and characterization of aggregates.
• Heat treatment promote protein polymerisation by intermolecular S-S bond formation.
• Molar mass distribution and conformation of polymers can be determined by A4F-MALLS.

## Graphical abstract

**Figure:**
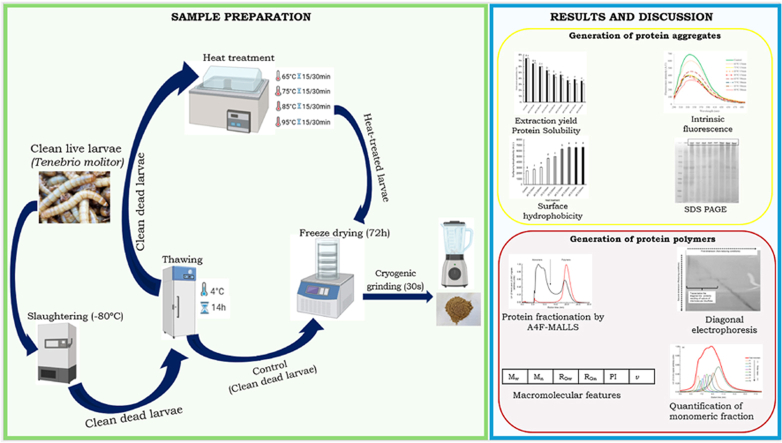

## 1 Introduction

Insects are proving to be a good alternative to conventional proteins due to their many advantages, particularly those related to the environment and nutrition. Insects have a shorter development time than traditional animals and their production requires little space for high-throughput production (van Huis et al., 2017). They also have a much higher feed conversion efficiency. For example, it requires 2.5, 5 and 10 kg of feed to produce 1 kg of chicken, pork and cow meat, respectively, compared to 1.7 kg for crickets or 1.69–1.89 kg for *Tenebrio molitor,* because insects extract their moisture requirement from food (Cadinu et al., 2020; Deruytter et al., 2022; Rumpold et al., 2013).

Nutritionally, compared to conventional proteins, insects are a high-quality protein source with a digestibility between 77 and 98 %, an essential amino acid score between 46 and 96 %, and an amino acid profile that meets the human nutritional requirements of the Food and Agriculture Organization (FAO) and World Health Organization (WHO) (Belluco et al., 2013). In addition, they are a good source of fibre, minerals (such as iron, zinc, potassium, sodium, calcium, phosphorus, magnesium, manganese and copper), vitamins (riboflavin, pantothenic acid, biotin and folic acid) and higher amounts of more essential fatty acids (linoleic and linolenic) (Belluco et al., 2013; De Castro et al., 2018). This composition makes insects suitable as a quality food for livestock and aquaculture (the farming of fish, crustaceans and other aquatic animals), pigs as well as dogs and cats, the latter of which they have good digestibility and palatability (Bosch et al., 2021; Hawkey et al., 2021; Kępińska-Pacelik et al., 2022; Smola et al., 2023).

Beyond the nutritional aspects, recent studies have revealed the technological potential of insect proteins by exploiting their solubility, gelling power, foaming power and emulsifying properties (Queiroz et al., 2023). For instance some authors observed that insect proteins presented excellent functional properties, close to or in some cases better than conventional sources such as whey, faba beans and yellow peas (Mishyna et al., 2019a; Stone et al., 2019).

To further explore these functionalities, which are strongly dependent on the physicochemical properties of insect proteins as well as their conformation, it is crucial to understand how the latter can be modified by the processing parameters used before and after protein extraction in order to focus on the desired properties and obtain high added-value ingredients (Gravel et al., 2020; Mishyna et al., 2021). Among the major practices in industrial food processing (such as the use of salt and pH alteration) (Lassé et al., 2015), the most used is heat treatment, which can be used to control microorganism growth and improve the shelf life of products. Thermal techniques, such as blanching, pasteurisation and ultra-high temperature (UHT), are commonly applied during food processing and aim to improve the colour, texture, quality, stability and safety of foods (Jo et al., 2018) and can be extended to alternative food sources, such as insects.

Some recent studies have suggested that heat treatments promote protein aggregation and oxidation and consequently may reduce the functional properties of insect proteins, such as solubility, oil absorption capacity, emulsification and foaming ability (Lee et al., 2019; Santiago et al., 2021; Zhang et al., 2022). Despite the interest raised by insect proteins in recent years, data on the impact of processes on their properties (structural and functional) are variable and remains limited (Gravel et al., 2020; Mishyna et al., 2021). In fact these studies that implement classic analytical protocols do not allow us to clearly differentiate the phenomena of aggregation (i.e. strict reinforcement of non-covalent bonds caused by protein denaturation and unfolding and exposition of internal hydrophobic groups) and polymerisation (strict reinforcement of intermolecular covalent bonds caused by protein oxidation) of proteins following thermal treatment. However, these phenomena probably do not have the same effects in terms of structural changes in proteins and therefore do not have the same effects on their functionalities.

It therefore appears essential to have new analytical approaches available today that allow us to characterise the extent of protein aggregation and/or polymerisation as a function of their size and quantity within a protein formulation, regardless of the production process. Field-flow fractionation (FFF), a concept first introduced by Giddings et al. (Giddings, 1966), is one of the most versatile separation techniques in the field of analytical separation sciences, capable of separating macromolecular colloidal and particulate materials, such as protein aggregates and polymers (Kumar et al., 2022; Nilsson, 2013; Runyon et al., 2014; Yohannes et al., 2011). AF4 is a sub-type of the larger family of FFF techniques (Giddings, 1993) that offers a useful alternative for protein aggregation/polymerisation characterization in comparison to size exclusion chromatography (SEC) since the method, apart from covering a very large size range and utilizing low shear rates (preserving fragile structures), can be performed under a broad range of buffer conditions (Liu et al., 2012). In the framework of the work of our laboratory, we have already been able to demonstrate the full effectiveness of this analytical methodology for the study of relationships between the aggregative and/or polymerised state of proteins and polysaccharides and their techno-functional properties (Aussenac et al., 2020; Chiaramonte et al., 2012; Lemelin et al., 2005).

**Fig. 1:**
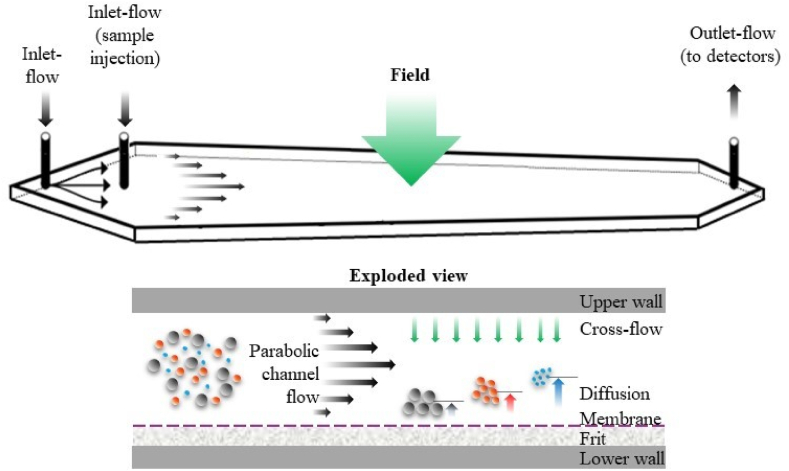
Structure of the AF4 channel and principle.

FFF theory applies almost directly to AF4, and it has been described in detail in numerous publications compiled in a review (Yohannes et al., 2011). Therefore, the fractionation principle is illustrated in Fig. 1. Separation takes place in an open channel consisting of two long, narrow blocks (i.e. upper and lower wall) bolted together with a spacer in between. Flow within this thin ribbon-like channel is laminar, with a pronounced parabolic flow profile that drives particle separation. The bottom block comprises a semi-permeable membrane supported by a frit. The membrane is permeable to the solvent but not to the analyte; this essential function is guaranteed by selecting the appropriate membrane pore size, expressed as a molecular-weight cutoff (MWCO) ranging from 1 to 100 kDa.

AF4 differs from symmetric flow in that channel flow is constantly lost through the single porous wall of the channel; thus, there is a decrease in the longitudinal flow velocity along the channel, compensated in part by the trapezoidal shape of the channel spacer (Litzen et al., 1991). Under laminar flow conditions, the channel flow has a parabolic profile, in which the velocity is greatest at the centre of the channel and lowest at the walls (Giddings, 1993). The downward force induced by the cross-flow is balanced by diffusional forces and differences in diffusion coefficients of analyte molecules (*D*) are the basis for the technique's selectivity. Lower masses with higher diffusion coefficients establish a steady state closer to the centre of forward flow and thus elute first, while higher masses traverse more slowly. Hence, AF4 fractionates samples based on differences in *D* [or conversely hydrodynamic size (rh)], and sample *Ds* can be directly determined from elution times(Litzen et al., 1991; Håkansson et al., 2012; Litzen, 1993; Magnusson et al., 2012). From measured *D* values the sample rh is obtained from the Stokes–Einstein equation (Einstein, 1905). The aim of our work was to determine the effect of heat treatment on the aggregation and polymerisation behaviour of mealworm protein extract. More specifically, the objectives were: (i) to determine the impact of heat treatment on aggregate formation by evaluating the modification of protein solubility, intrinsic fluorescence, surface hydrophobicity and sodium dodecyl sulphate polyacrylamide gel electrophoresis (SDS-PAGE) profiles; and (ii) to quantify and characterise polymer formation by applying a novel analytical approach (asymmetric flow field-flow fractionation coupled with multi-angle laser light scattering) to determine the impact of heat treatment on the macromolecular (polymer to monomer ratio and molar masses and radii of gyration distributions) and conformational (polydispersity index and shape) characteristics of protein polymers.

## 2 Materials and methods

### 2.1 Materials

The larvae (*Tenebrio molitor*) used in this study were provided by Ynsect (Evry, France). Urea (electrophoresis grade), sodium dodecyl sulphate (SDS), sodium phosphate dibasic and monobasic, trichloroacetic acid (TCA), sodium hydroxide (NaOH), dithiothreitol (DTT) and bovine serum albumin (BSA) were purchased from Merck (Saint Quentin Fallavier, France). The Bis-ANS (acide 4,4′-dianilino-1,1′-binaphthyl-5,5′-disulphonique, dipotassium salt) and the reagents used for SDS-PAGE analysis, such as protein marker, electrophoresis buffers (NuPAGE LSD Sample Buffer and NuPAGE MOPS SDS Running Buffer), reducing agent (NuPAGE Sample Reducing Agent), gels (NuPAGE Bis-tris 4–12 % polyacrylamide), antioxidant (NuPAGE antioxidant) and staining solution (SimplyBlue SafeStain) were purchased from Fisher Scientific (Waltham, Massachusetts, USA).

### 2.2 Sample preparation and heat treatment

**Fig. 2:**
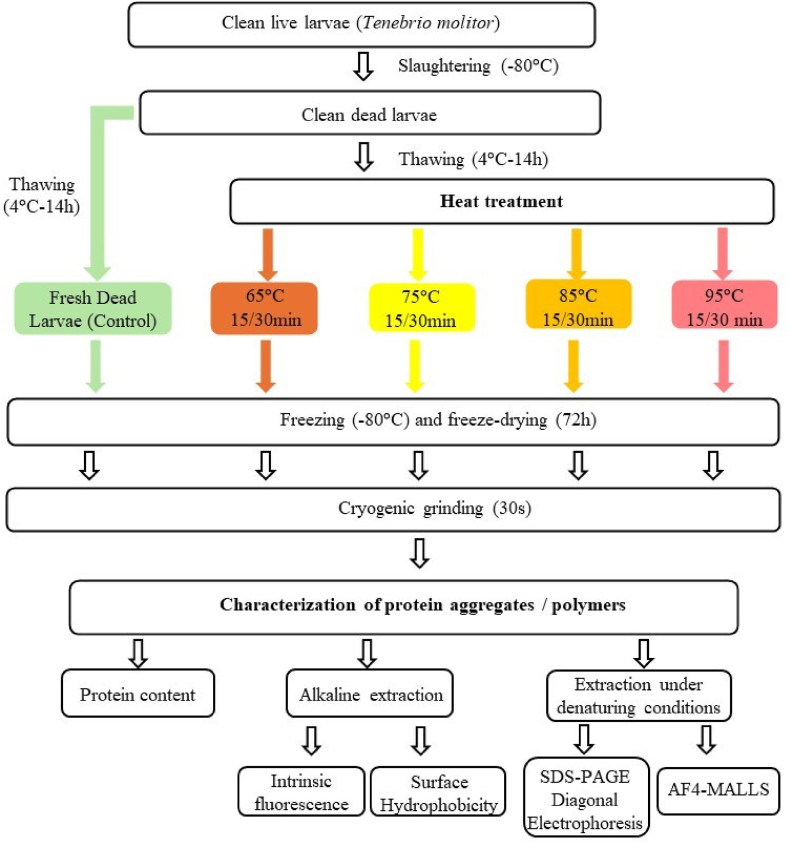
General schema of sample preparation, extraction and protein characterisation.

*Tenebrio molitor* larvae were first starved for 24 h to empty their digestive tracts, sieved and sorted to remove frass residue and obtain clean fresh larvae (without pupae). They were then placed at −80 °C to kill them quickly and avoid browning (Fig. 2). Before heat treatment, the larvae were placed in the refrigerator (14 h) to thaw them and facilitate treatment. Three stainless steel filters (7.5 × 9.5 × 7.5 cm) were filled with 50 g of larvae each and then immersed for 15 or 30 min in a water bath (WNB 22, Memmert, Germany) at different temperatures (65, 75, 85 and 95 °C). After heat treatment, the larvae (untreated and heat-treated) were placed at −80 °C for a rapid decrease in temperature and to stop the treatment. Before grinding, the larvae were freeze-dried (VirTis 8L Benchtop Freeze Drye) for 72 h and then batches of 50 g were prepared. Insect meals were prepared by cryogenic grinding and then carried out (Moulinex, 100W, DPA141) for 30 s in different batches.

### 2.3 Insect meals dry matter content

The dry matter content of different insect meals was determined by drying 3 g of meal at 110 °C for 1 h and then at 80 °C for 48 h. The dry matter content was calculated as follows (Eq. (1)):

(Eq. 1) $$Dry\;matter\;\left(\%\right)=\left(1-\frac{Mi-Mf}{Mi}\right)\times 100$$

where *Mi* and *Mf* are the initial and final weights of sample before and after drying, respectively.

### **2.4** Insect meals protein **content**

The protein content of insect meals was determined following the Dumas method (method- AOAC 7024) using a total nitrogen analyser (model FP528, Leco France, France). To determine the protein content of insect meals, a conversion factor of 5.6 was applied to the total nitrogen content. Analyses were carried out on dry matter (80–100 mg) in triplicate.

### 2.5 Dried mealworm larvae protein (DMLP) extraction

#### 2.5.1 Alkaline extraction of DMLP

A 25-mL volume of ultrapure water was added to 0.5 g of insect meals. The mixture was homogenised by vortexing, and the pH was quickly adjusted to 12 using an aqueous NaOH solution (0.1 or 1 M). The mixture was placed on a rotary shaker (MX-RD Pro) for 1 h at 40 rpm. The supernatant was collected after centrifugation (10,000*×g*, 10 min, 4 °C), and the pellet was oven dried (50 °C, 72 h). The supernatant was frozen (−80 °C) and lyophilised (72 h) before protein quantification (Dumas method). Yiled and solubility was determined as follows (Eqs. (Eq. 2), (Eq. 3))):

(Eq. 2) $$Yield\;\left(\%\right)=\frac{Mi-Mc}{Mi}\times 100$$

(Eq. 3) $$Solubility\;\left(\%\right)=\frac{Mi\times Xi-Mc\times Xc}{Mi\times Xi}\times 100$$

where *Mi* and *Mc* are the weights (dry matter, in grams) of the initial sample and the pellet, and *Xi* and *Xc* are the protein contents (%) of the initial sample and the pellet, respectively.

#### 2.5.2 Extraction of DMLP under denaturing conditions

To determine the molecular weight distribution of the proteins, another extraction was carried out in phosphate buffer [0.1 M, pH 7 contained 2 % (w/v) SDS and 8 M urea]. Briefly, in a polypropylene tube, 10 mL of extraction buffer was mixed with 0.5 g (DM) of insect meal and heated at 50 °C for 1 h in a water bath and vortexed after 30 min. The solutions were subjected to ultrasonic treatment (20 kHz, 120 W, 50 % input, 1 min) (Fisher Scientific, Model FB120) and then centrifuged (10,000*×g*, 10 min, 4 °C). The supernatants were kept at 4 °C for future analysis. The pellets were washed with ultrapure water (30 mL) by stirring (40 rpm) and then centrifuged (10,000*×g*, 10 min, 4 °C) to remove the excess urea. This operation was replicated three times. The resulting pellets were oven dried at 50 °C for 72 h. The percentage of protein extracted (i.e. protein solubility) was determined as described for water extraction.

### 2.6 DMLP protein characterisation

#### 2.6.1 Intrinsic fluorescence of alkaline soluble DMLP

Protein intrinsic fluorescence was measured using the method described by Boukil et al. (2022) with some modifications. A 0.05 % (w/v) protein extract was prepared in a phosphate buffer (PBS 0.1 M, pH 7), and the intrinsic fluorescence (emission spectrum) was measured between 290 and 500 nm (slit width:3 nm) using a spectrofluorometer (SpectraMax M2, Molecular Devices, San Jose, USA) with an excitation wavelength of 280 nm.

#### 2.6.2 Surface hydrophobicity of alkaline soluble DMLP

Protein surface hydrophobicity was determined using the protocol developed by Jiang et al. (2021) with modifications. A protein extract (0.005, 0.01, and 0.02 % w/v) was prepared in phosphate buffer (0.1 M, pH 7), and 20 μL of bis-ANS (8 mM in 0.1M phosphate buffer, pH 7) was used to obtain a final volume of 3 mL. The solutions were stirred and incubated in the dark (10 min), and fluorescence was measured at excitation/emission wavelengths (380/485 nm). The slope of the linear regression of the fluorescence intensity versus the protein concentration represented the protein surface hydrophobicity [expressed in arbitrary units (A.U.)].

#### 2.6.3 Sodium dodecyl sulphate polyacrylamide gel electrophoresis (SDS-PAGE)

Proteins from supernatants extracted in denaturing conditions were precipitated with TCA (10 mL at 12 % w/v) and recovered after centrifugation (10,000*×g*, 10 min, 4 °C). This step was repeated twice to remove excess urea. The pellets obtained were then frozen at −80 °C. After freeze-drying, the protein content of the residue was determined (Dumas method).

SDS-PAGE was performed under non-reducing and reducing conditions. Twenty microlitres of each protein sample (2 mg/mL) was added to 20 μL of loading buffer. This loading buffer contained 25 % (v/v) NuPAGE LDS sample buffer (Coomassie G250, glycerol, pH 8.4) and 75 % (v/v) ultrapure water under non-reducing conditions. For the reducing condition, 25 % (v/v) NuPAGE LDS sample buffer, 65 % (v/v) ultrapure water and 10 % (v/v) NuPAGE sample reducing agent (500 mM DTT) were used. After heating at 70 °C in a water bath for 10 min, 20 μL of each sample (protein extract + loading buffer) was loaded into the gel wells (NuPAGE Bis-tris 4–12 % polyacrylamide, 10 wells, 1.5 mm thickness). Then, gels were placed in the Mini Gel Tank (Thermo Fisher Scientific), and protein separation was carried out at 190 V for 1 h using the migration buffer (NuPAGE MOPS SDS running buffer). Gels were then washed 3 times with ultrapure water (5 min) and stained (Coomassie G-250, SimplyBlue SafeStain) for 1 h at room temperature. The gels were then washed for 1 h with water to remove excess dye and reveal the bands.

#### 2.6.4 Determination of the monomeric composition of protein polymers

The proteins were first separated under non-reducing conditions (first dimension), as described in the previous section (2.6.2) but using a NuPAGE Bis-tris 4–12 % polyacrylamide, 9 wells, 1 mm thickness gel. After this, one loading well of the gel was collected, ground and incubated for 16 h in 2 mL of reducing solution [0.1M, pH 7 contained 2 % (w/v) SDS and 8M urea, 1 % (w/v) DTT]. After incubation, the solution was centrifuged (10,000×*g*, 10 min, 4 °C) and filtered through a syringe filter of regenerated cellulose (porosity 0.45 μm). The filtrate was then concentrated using a centrifugal filter unit (cut-off 3 kDa, 4019×*g*, 30 min, 4 °C). The resulting protein extract (50 μL) was mixed with 50 μL of loading buffer, and migration was performed as in section 2.6.2.

#### 2.6.5 Diagonal electrophoresis

Diagonal electrophoresis is a sequential non-reducing analysis followed by a reducing gel analysis procedure that can allow the unbiased identification of proteins that form inter-molecular disulphides (Eaton, 2006).

The proteins were first separated under non-reducing conditions (first dimension), as described in the previous section (2.6.2) but using a NuPAGE Bis-tris 4–12 % polyacrylamide gel with 9 wells and 1-mm thickness. Then, the migration lane (zone of interest) of the gel was cut vertically and incubated in 15 mL of a reducing solution [25 % (v/v) NuPAGE LDSsSample buffer, 70 % (v/v) ultrapure water and 5 % (v/v) 2-mercaptoethanol] for 1 h (2 × 30 min). After reduction, the band obtained was placed on top of a gel (NuPAGE Bis-tris 4–12 % polyacrylamide, 10 wells, 1.5 mm thickness) to perform the second-dimension separation (190 V, 1 h). Gels were then washed 3 times with ultrapure water (5 min) and stained (Coomassie G-250, SimplyBlue SafeStain) for 1 h at room temperature. The gels were then washed for 1 h with water to remove excess dye and reveal the bands.

#### 2.6.6 Asymmetrical flow field-flow fractionation (AF4)

The protein molecular weight distribution was determined by AF4 from the denatured protein extracted. Lyophilised protein extracts were solubilised in 0.1 M phosphate buffer, 2 % SDS (w/v), and 8 M urea (pH 7.0, 5 mg protein/mL). Protein extracts were filtered through a syringe filter of regenerated cellulose (porosity 0.45 μm). The AF4/MALLS system consisted of an eclipse3 F system (Wyatt Technology, Santa Barbara, CA, USA), equipped with a regenerated cellulose membrane (cut-off, 10 kDa, Wyatt Technology, Santa Barbara, CA, USA) combined with a UV detector (214 nm, Agilent 1200, Agilent Technologies, Waldbronn, Germany), a MALLS detector (Dawn multiangle Heleos TM, Wyatt Technology Corporation), and an interferometric refractometer (Optilab rEX, Wyatt Technology Corporation). A 0.1 M phosphate buffer containing 0.1 % (w/v) SDS (pH 7), was used as the elution mobile phase. Thirty microlitres of each sample was injected and separated using a method composed of two modes (focus and elution). The focus time was 4 min with a constant cross-flow rate of 2 mL/min. The elution was divided into two phases, the first of which was linear for 14 min, with a cross-flow rate decreasing exponentially from 3.0 to 0.0 mL/min, and the second at a constant cross-flow rate of 0.0 mL/min for 14 min. MALLS photodiode coefficients were normalised using the bovine serum albumin (BSA) monomer as reference. Calculations of molecular weight number-average (M_n_), weight-average (M_w_) and z-average (M_z_) and mean square radius number-average (R_Gn_), weight-average (R_Gw_) and z-average (R_Gz_) were performed using ASTRA 4.72 and Corona software (Wyatt Technology, Santa Barbara, CA, USA). Zimm extrapolation (first order) (KC/Rθ) was used to calculate M and R_G_. The value for dn/dc was measured as described by Wittgren et al. (2000). A value of 0.250 mL/g was used as a refractive index increment (dn/dc) for the wheat proteins. To determine the monomeric-to-polymeric ratio, the UV signal (214 nm) was integrated for each protein region (M < 10^5^ g/mol and M ≥ 10^5^ g/mol) using ASTRA 4.72 software.

### 2.7 Deconvolution of monomeric UV signals

To determine the percentage of the different monomeric fractions fractionated by AF4, we applied a specific deconvolution method to the fractograms using Lorentzian functions to obtain the calculated areas for the identified fractions. The deconvolution method was developed using ORIGIN 5.0 software professional (OriginLab Corporation) with the following Lorentz function (Eq. (4)) evaluated in a previous work of our lab (Kadri et al., 2023):

(Eq. 4) $$y=y0+\frac{2A}{\pi }\;\frac{w}{4{\left(x-x_{c}\right)}^{2}+w^{2}}$$

where *x*_*c*_ represents the value of the fit mode wavenumber, *A* the area of the peak and *w* the width at mid-height. We take an iteration number equal to 100.

### 2.8 Statistical analysis

An analysis of variance (ANOVA) was carried out with Excel software (Microsoft, Redmond, Washington, USA, version 2412). All results were performed in triplicate. Differences between samples were determined using Tukey's post-hoc test (a probability level of 0.05) using XLSTAT (26.4.2 version, Paris, France).

## 3 Results and discussion

### 3.1 Dry matter and protein content of insect meals

The dry matter and protein content of the insect meals used in this study are shown in Table 1. The dry matter percentages ranged from 97.15 to 98.33 %. Protein contents varied from 48.84 to 51.47 %, which is very close to the results published in previous studies with protein contents (Rumpold et al., 2013; Yoo et al., 2019; Zhao et al., 2016).

**Table 1:** Dry matter and protein content of insect meals.

Sample treatment	Dry matter (g/100g meal)	Protein content (g/100g DM)
Control	97.15 ± 0.02(a)	51.47 ± 0.27(a)
65 °C/15min	98.33 ± 0.06	50.68 ± 0.55
65 °C/30min	97.94 ± 0.19	48.98 ± 0.75
75 °C/15min	97.93 ± 0.10	48.96 ± 0.40
75 °C/30min	97.78 ± 0.11	49.14 ± 1.69
85 °C/15min	97.73 ± 0.05	49.10 ± 0.60
85 °C/30min	97.88 ± 0.07	48.36 ± 0.55
95 °C/15min	97.77 ± 0.04	48.84 ± 0.41
95 °C/30min	97.99 ± 0.03	49.22 ± 0.99

(a) Mean ± standard deviation (n = 3).

### 3.2 Impact of heat treatments on the major physicochemical properties of DMLP: generation of protein aggregates

#### 3.2.1 Alkaline extraction of DMLP

**Fig. 3:**
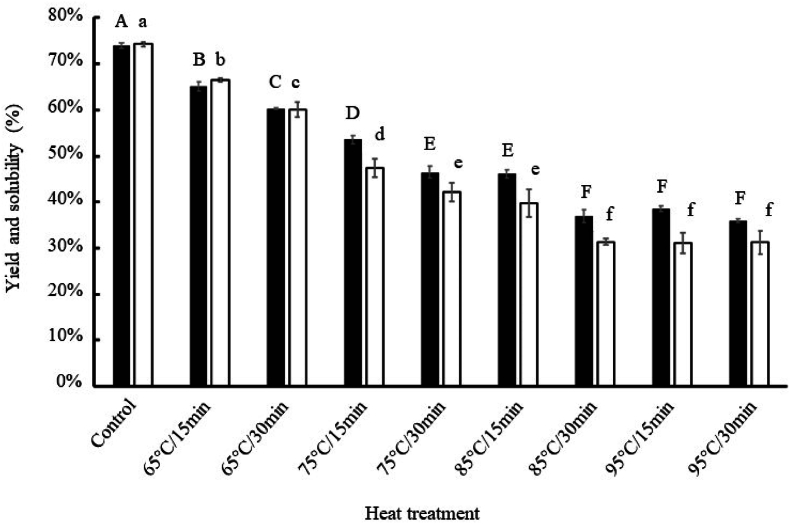
Extraction yield (black bars) and protein solubility (white bars) in alkaline conditions (pH 12.0) after heat treatment. Different letters indicate a significant difference at p < 0.05.

Solubility represents an important indicator of the functional properties of proteins (Amagliani et al., 2017; Yousefi et al., 2022). The results of extraction yield and protein solubility are shown in Fig. 3. Increasing temperature and/or time caused a significant decrease in both the extraction yield and protein solubility (from 74 to 37 % and from 74.5 to 31 % for yield and solubility, respectively); this loss in yield and solubility reached its maximum under experimental conditions of 85 °C/30 min. Our observations are consistent with some previous studies that have reported, in particular, a decrease in the solubility of *Tenebrio molitor* proteins after thermal treatment from 75 to 95 °C (Lee et al., 2019), between 80 and 140 °C (Buβler et al., 2016), or after microwave treatment (800 W, 10 min) (Zhang et al., 2022). The relative impact of temperature and heat treatment duration on protein solubility was analysed using a two-factor ANOVA (Table 2). The duration of heat treatment as well as the applied temperature had very significant effects on protein solubility under alkaline conditions (*p*-values of 2.05 × 10^−18^ and 6.32 × 10^−9^ for temperature and time, respectively); the applied temperature had a major effect (mean squares ratio 1213.43/149.10 = 8.14) (Table 2). In addition, the two-factor ANOVA revealed a large significant interaction between these factors (*p*-value = 3.63 × 10^−5^).

**Table 2:** Effects of time and temperature on the solubility of DMLP (two-factor ANOVA).

Variation source	Degrees of freedom (df)	Sum of squares (SS)	Mean squares (MS)	Probability (*p*-value)	F
Time (t)	1	149.10	149.10	6.32 × 10^−9^	123.18
Temperature (T)	3	3640.29	1213.43	2.05 × 10^−18^	1002.43
Interaction (T × t)	3	60.22	20.07	3.63 × 10^−5^	16.58
Error	16	19.38	1.21		

The modifications of the solubility of DMLP under the effect of heat treatment can have, as their main origin, an acceleration of the aggregation phenomena of the present proteins (Wang et al., 2010). According to some authors (Pelegrine et al., 2005; Timilsena et al., 2016), for a constant pH and beyond their denaturation temperature, the solubility of proteins decreases rapidly due to a significant reduction in protein–water interactions caused by the exposure of their hydrophobic groups. We will be able to validate this phenomenon by evaluating the structural and conformational modifications of the proteins (i.e. fluorescence properties of DMLP).

#### 3.2.2 Fluorescence of alkaline soluble DMLP

For fluorescence measurements of proteins (DMLP), we chose to perform an alkaline extraction (aqueous solution at pH 12.0). Despite that some extraction yields were low (Fig. 2), this method is similar to that used in the literature. It better preserves the protein structures (compared to extraction procedures using denaturants), and the pH used corresponds to a region of maximised solubility of insect proteins (Anusha et al., 2023; Gkinali et al., 2022; Jeong et al., 2021).

##### 3.2.2.1 Intrinsic fluorescence of alkaline soluble DMLP

Intrinsic fluorescence is a commonly used method for monitoring changes in the tertiary structure of proteins when they have been subjected to modifications during larval processing (Garidel et al., 2008; Malik et al., 2019). The conformational modifications of DMLP were assessed by monitoring changes in the intrinsic fluorescence emission of the tryptophan indole group between 290 and 500 nm.

**Fig. 4:**
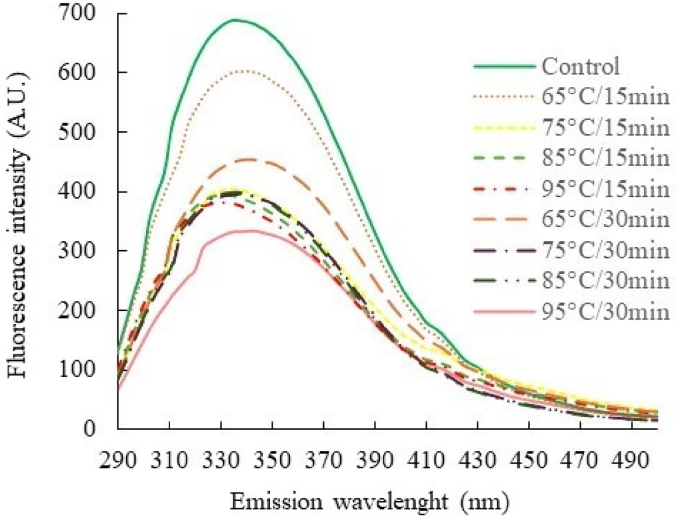
Intrinsic fluorescence of alkaline soluble DMLP after heat treatment. Fluorescence excitation wavelength: 280 nm.

The different results obtained are presented in Fig. 4 and illustrate the effects of heat treatments on the tertiary structure of the extracted proteins. Thus, the heat treatments implemented in our study caused a very significant decrease (i.e. reduction of more than half) in the fluorescence intensity of proteins. Furthermore, the fluorescence intensity seemed to be more impacted by a high temperature (i.e. above 85 °C) regardless of the treatment time applied. Some studies have shown similar results. A decrease in the fluorescence of *Tenebrio molitor* proteins was observed after thermal treatment at 20–140 °C for an extract at pH 4 (Buβler et al., 2016) and for another silkworm pupae extract heated to 60, 80 and 100 °C (He et al., 2021). Other studies have also shown that the fluorescence of *Tenebrio molitor* larvae proteins can be reduced after treatment with microwaves at 850 W for 10 min (Zhang et al., 2022) and high hydrostatic pressure at 600 MPa (Boukil et al., 2022). Furthermore, *Protaetia brevitarsis* proteins treated with gamma-ray or electron beam irradiation showed a similar trend (Hoon Lee et al., 2024).

##### 3.2.2.2 Surface hydrophobicity of alkaline soluble DMLP

Surface hydrophobicity is the measure commonly used to evaluate the modification of protein conformation. ANS binds to the hydrophobic sites of protein, reflecting their capacity to undergo structural modification (Chen et al., 2018; Hayakawa et al., 1985).

**Fig. 5:**
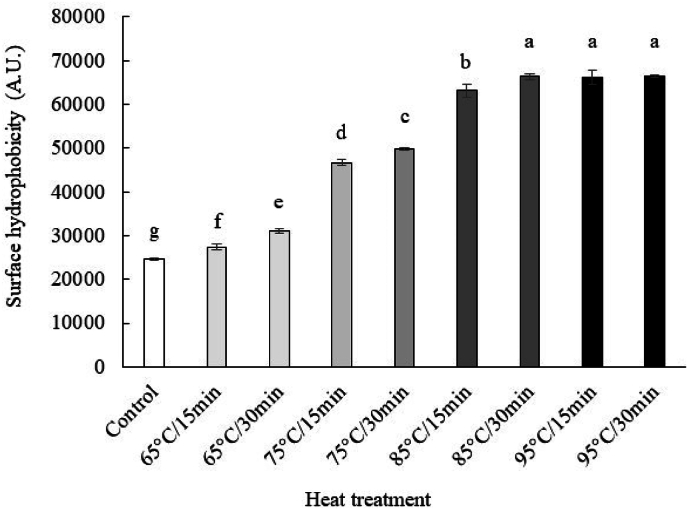
Surface hydrophobicity of alkaline soluble DMLP after different heat treatment. Different letters indicate a significant difference at p < 0.05.

The changes in the surface hydrophobicity of DMLP solutions are shown in Fig. 5. The surface hydrophobicities (A.U) of the control and heat-treated mealworm larvae proteins at 65 °C were comparable, even though they were statistically different (*p* < 0.05), with values ranging from 24,611 to 31,060. However, the surface hydrophobicities of mealworm larvae protein extracts receiving heat treatments from 75 to 95 °C (from 46,703 to 66,500) were significantly (*p* < 0.05) greater than those of the control (24,611). In addition, similar surface hydrophobicity values were obtained for heat treatments from 85 °C for 30 min to 95 °C for 30 min (*p* < 0.05).

The results showed that heating, by reducing intrinsic fluorescence and increasing the surface hydrophobicity of the proteins, altered their tertiary structure by promoting aggregate formation. Temperature caused proteins to unfold, exposing the hydrophobic groups and leading to the formation of insoluble aggregates; these observations are consistent with those of previous works (Meng, 2002; Wang et al., 2014).

#### 3.2.3 Extraction of DMLP under denaturing conditions after heat treatment

Urea belongs to a class of compounds known as chaotropic denaturants, which unravel the tertiary structure of proteins by destabilising internal, non-covalent bonds between atoms (Das et al., 2008; Wei et al., 2010). Even if proteins can be denatured by urea through several processes (i.e. direct and indirect process) (Bennion et al., 2003), their solubility generally increases after treatment because of the dissociation of the protein aggregates.

**Fig. 6:**
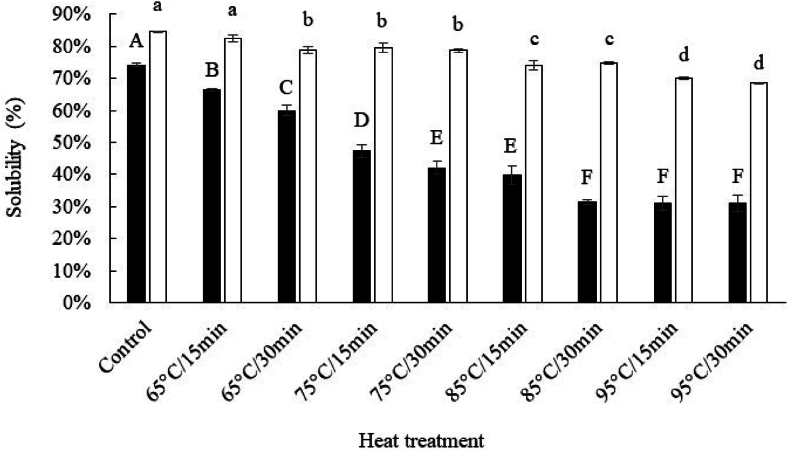
Solubility of DMLP after different heat treatments in alkaline conditions (pH 12.0) (black bars) and in 0.1 M PBS at pH 7, 2.0 % SDS and 8 M urea (denaturing conditions) (white bars). Different letters (capital and/or lowercase) indicate a significant difference at p < 0.05.

The DMLP extraction results obtained after using a high urea concentration (8 M) in the presence of SDS (2 % w/v) are presented in Fig. 6 and are compared to those previously obtained from an extraction under alkaline conditions (pH 12.0). The addition of urea in the presence of SDS significantly increased (p < 0.05) protein solubility in all samples (from +10.24 to +37.36 % for control and heat-treated samples, respectively). The increase in protein solubility was more significant when the temperature was high. For 65, 75, 85 and 95 °C, the average difference in protein solubility was +17, +34, +39 and + 38 %, respectively. At the same time, the time of exposure to heat treatment (i.e. 15 min *vs.* 30 min) had no significant impact (*p* > 0.05) on this phenomenon.

However, despite the denaturing action of urea (i.e. disruption of non-covalent protein–protein interactions), the solubility of some heat-treated samples (especially 85 and 95 °C) remained significantly (p < 0.05) lower than that of the control (Fig. 6). This demonstrates that other types of protein–protein interactions (i.e. covalent bonds not sensitive to the action of urea and SDS) are responsible for this solubility deficit. In preliminary tests (data not shown), a very large part of this protein material can be solubilised by adding a reducing agent [for example DTT, 1 % (w/v)] to the extraction solution (0.1 M PBS, pH 7; 2 % SDS; 8M urea). These observations/hypotheses were confirmed through protein analysis using SDS-PAGE.

**Fig. 7:**
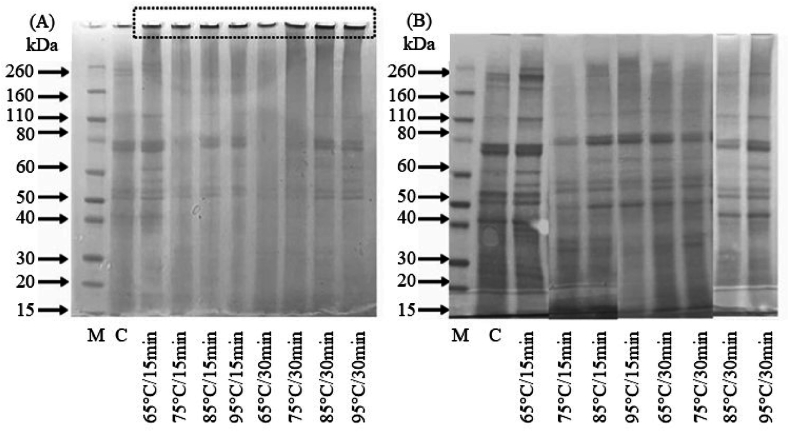
SDS-PAGE separation of DMLP after different heat treatments (A) under non-reducing conditions and (B) under reducing conditions. Protein markers (M line) with corresponding molar masses in kDa (black arrows), Control (C line) and heat (other lines).

The results of these analyses are presented in Fig. 7A and B for SDS-PAGE separations under non-reducing conditions (without DTT) and reducing conditions (with DTT), respectively. Under non-reducing conditions (Fig. 7A), the different lines (control and heat-treated samples) were characterised by a certain number of proteins in the monomeric state whose bands were identifiable in terms of molar mass (bands between 20 and 90 kDa) but also by the presence of oligomeric forms (diffuse traces with M_w_ > 110 kDa). These oligomeric forms disappear significantly when implementing reducing conditions (Fig. 7B), thus demonstrating the existence of covalent disulphide-type bonds (S-S) within these protein assemblies.

At the same time, under non-reducing conditions (Fig. 7A), a significant amount of protein was detected in the loading wells of the acrylamide gel, indicating the presence of large protein polymers (Mw > 260 kDa). The staining intensity of these loading wells (marked with a dotted frame in Fig. 7A), which is proportional to the amount of proteins present, clearly increased with the intensity of the heat treatment applied (from 65 to 95 °C).

Under reducing conditions (Fig. 7B), this staining was significantly reduced for most samples, suggesting that the proteins present within these polymeric forms were associated with S-S covalent bonds that were completely dissociable by the reducing agent. These results are consistent with observations made by some authors when *Tenebrio molitor* proteins were heated or subjected to other treatments, such as high hydrostatic pressure (Santiago et al., 2021; Zhang et al., 2022; Boukil et al., 2022; Buβler et al., 2016; Mishyna et al., 2019b). However, unlike our work, these authors do not, in any case, strictly distinguish between protein aggregation (formation of non-covalent bonds) and/or protein polymerisation (formation of covalent bonds) phenomena.

### 3.3 Impact of heat treatments on DMLP oxidation: generation of protein polymers

#### 3.3.1 Fractionation of DMLP by A4F-MALLS under denaturing conditions

The AF4 method, the principles of which were detailed in the materials and methods section, is a particularly versatile analytical technique for separating particles according to size, capable of fractionating the biological polymers and/or aggregates without shear and adsorption constraints thanks to the absence of a stationary phase.

In our work, the analytical protocol used allows us to separate, characterise and quantify the different protein fractions of DMLP without size and/or conformation limitations and without the need for reference to conventional calibration methods (based on elution times) but using the light scattering (LS) properties of the particles.

**Fig. 8:**
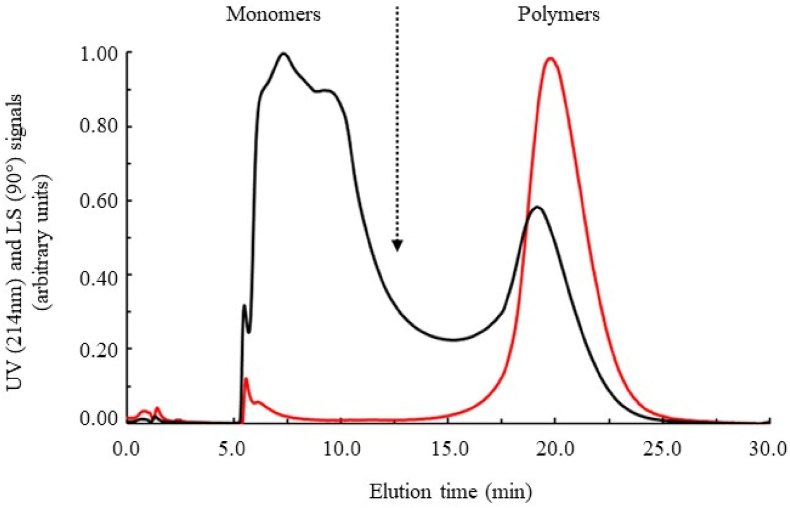
Asymmetrical flow field-flow fractionation (AF4) profiles of DMLP treated at 95 °C for 15 min. UV detection at a 214-nm signal (black line) and light scattering signal at 90° (red line). The dotted line materialises the theoretical limit between fractions of monomeric proteins *vs.* polymeric proteins based on the calculation shown in section 2.6.5.

As illustrated by the results of the sample used (heat treatment 95 °C/15 min) in Fig. 8 (UV signal vs. LS signal), the developed methodology allows highly significant discrimination of the extracted protein fractions. The light scattering and UV signals did not coincide, which was an effect of the high polydispersity of the protein sample (significant presence of oligomers and polymers). This fractogram also illustrated the remarkable power of light scattering detection to study the high molar mass fraction (M). Thus, despite the presence of very low amounts of very high molar mass species, a MALLS signal was clearly visible.

**Fig. 9:**
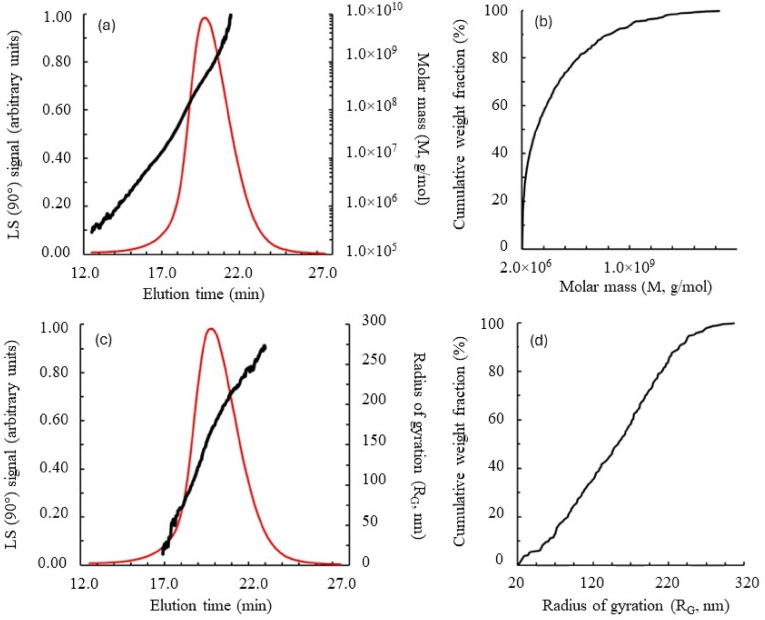
Hydrodynamic parameters of DMLP (Cf. Fig. 1). (a) Molar mass (M) as a function of elution volume; (b) cumulative distribution of the molar mass; (c) radius of gyration (R_G_) as a function of elution volume; and (d) cumulative distribution of the radius of gyration. Light scattering signal at 90° (red line).

Fig. 9 shows an example of the completed results of the AF4-MALLS experiment on the polymeric proteins in this insect meal. In Fig. 9(a–c), the molar masses (M) and the radii of gyration (R_G_) of the polymeric proteins calculated from initial curves of the scattering functions are plotted as a function of the elution time, respectively. By including the concentration, which was measured with UV detection for each of the eluates investigated, both the molar mass and the radius of gyration distribution were calculated, as shown in Fig. 9 (b,d, respectively). The data were used to determine the characteristic average values of M_n_, M_w_, R_Gn_ and R_Gw_ by means of their definition relationships (Wyatt, 1991). For our experiments, these results are compiled and discussed in more detail later (Table 3).

**Table 3:** Protein composition (monomers vs. polymers) in heat-treated DMLP.

Protein State	Protein fractions(b)	Heat treatment(a)				
		Control	65 °C/15 min	75 °C/15 min	85 °C/15 min	95 °C/15 min
Non reduced	Monomers	98.00 ± 0.01^a^	95.83 ± 0.06^d^	89.20 ± 0.10^e^	72.30 ± 0.36^f^	65.40 ± 0.10^g^
	Polymers	2.00 ± 0.01^n^	4.17 ± 0.06^k^	10.80 ± 0.14^j^	27.70 ± 0.36^i^	34.60 ± 0.07^h^
	*P/M ratio*(c)	*0.02*	*0.05*	*0.12*	*0.38*	*0.53*
Reduced	Monomers	98.07 ± 0.06^a^	97.83 ± 0.12^a^	97.73 ± 0.15^a^	97.23 ± 0.06^b^	96.53 ± 0.06^c^
	Polymers	1.93 ± 0.06^n^	2.17 ± 0.12^n^	2.27 ± 0.15^n^	2.77 ± 0.06^m^	3.47 ± 0.06^l^
	*P/M ratio*	*0.02*	*0.02*	*0.02*	*0.03*	*0.04*

Different letters within a column or/and line indicate significantly different values (p < 0.05).

(a) Mean ± standard deviation (n = 3).

(b) As defined in Fig. 9. Amount (%) estimated from the calculation of chromatographic surfaces (UV signals).

(c) Polymer to monomer ratio.

These original results demonstrate that proteins extracted from DMLP can be fractionated, quantified and characterised by AF4-MALLS-UV regardless of their degree of association. A significant amount of the extracted proteins existed in the form of oligomers and polymers, characterised by very high molar masses (from 10^5^ to 10^10^ g/mol) and radii of gyration (from 20 to 260 nm) (Fig. 9a–c), even if the solubilisation conditions used here were strongly denaturing (i.e. presence of urea and SDS). In full agreement with the molar mass distribution presented in Fig. 8, an overall separation of monomeric (R_G_ < 10 nm) *vs.* polymeric (R_G_ > 10 nm) forms is shown in Fig. 8 (black arrow).

These observations, even if they agree with the results obtained during SDS-PAGE analyses under non-reducing conditions (Fig. 7A), illustrate the analytical limits of these classical approaches (i.e. a significant amount of uncharacterised protein material present in the gel deposition wells). These limits seriously hamper the characterisation of the association state of these proteins. In fact, the method developed here is a rapid procedure for the measurement of the molar mass distribution (MMD) and the radius of gyration distribution (RGD) of DMLP, characteristics which can be determinant for the prediction of their functional properties (Queiroz et al., 2023).

#### 3.3.2 Impact of heat treatment on the molecular mass and size distribution of DMLP

**Fig. 10:**
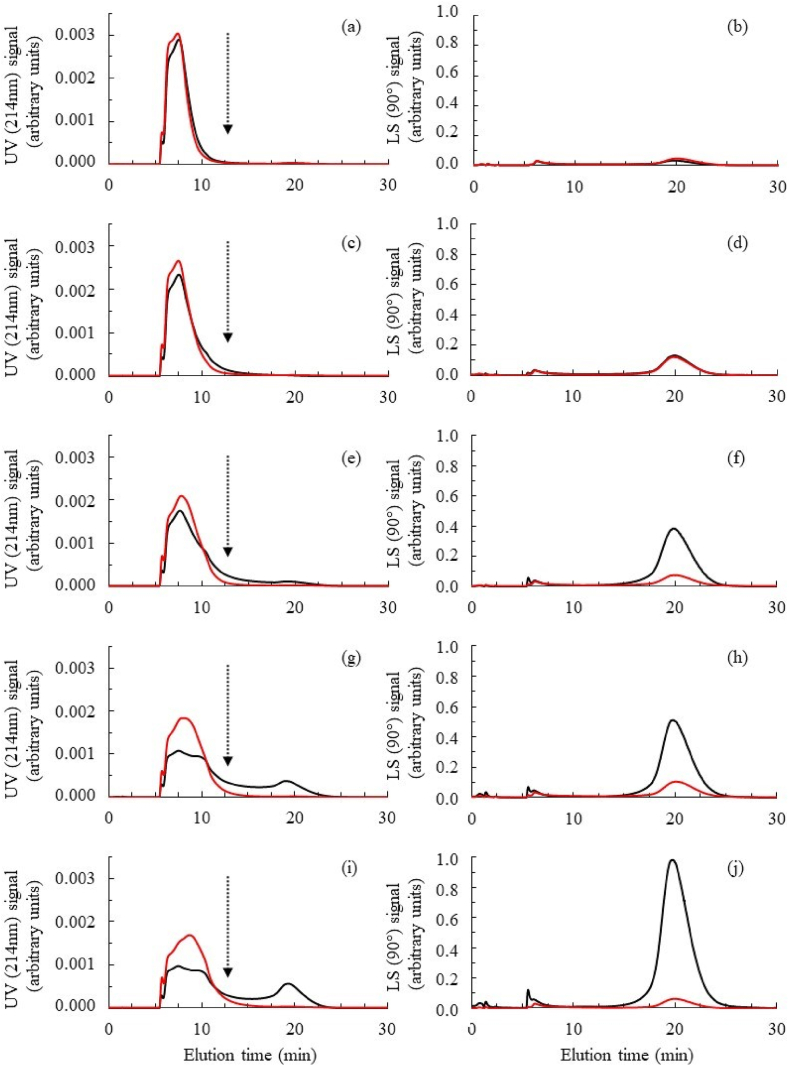
Asymmetrical flow field-flow fractionation (AF4) profiles of DMLP under non-reduced (black line) and reduced conditions (red line). UV detection signal for (a) control, (c) 65 °C/15 min, (e) 75 °C/15 min, (g) 85 °C/15 min and (i) 95 °C/15 min, LS (90°) signal for (b) control, (d) 65 °C/15 min, (f) 75 °C/15 min, (h) 85 °C/15 min and (j) 95 °C/15 min. The dotted line materialises the theoretical limit between the fractions of monomeric proteins *vs.* polymeric proteins.

As part of our study, this analytical approach (AF4-MALLS-UV) was used to highlight the effects that different heat treatments have on the state of association (MMD and RGD) of insect meal proteins. First, the increase in temperature applied to insect meal induced the formation of protein oligomers and polymers by mobilising the different monomeric fractions initially present in the extracts (Fig. 10 and Table 3).

Thus, when almost all the proteins (98.00 %) present in the control DMLP (not heat-treated) were characterised by their monomeric state (Fig. 10a and b) (Table 3), these monomeric forms only represented 65.40 % for the 95 °C treatment (Fig. 10i and j) (Table 3). In the context of our experiment, even when the formation of these protein polymers was detectable from 65 °C (4.47 %) (Table 3 and Fig. 10c and d) due to the very high sensitivity of MALLS detection, the quantities were only really significant (based on UV signal) for heat treatments exceeding 75 °C (>10 %) (Table 3 and Fig. 10e–j).

A first characterisation of the distribution of molecular masses of proteins was defined by the ratio of polymeric to monomeric forms (P/M ratio). This varied greatly depending on the intensity of the applied heat treatment, from 0.02 to 0.53 for the control meal and the treatment at 95 °C (Table 3).

Second, the macromolecular features of polymeric proteins themselves evolved under the effect of the applied thermal treatments. Thus, as shown by the data collected in Table 4, all parameters characterising the polymers present (M_w_, M_n_, R_Gw_ and R_Gn_) evolved according to the temperature applied to the insect meals. The weight-average molar mass (M_w_) thus increased from 18,695.57 × 10^4^ to 37,113.36 × 10^4^ g/mol ( × 2) for temperatures increasing from 65 to 95 °C. The same phenomenon was observable from the values of M_n_ [from 39,914.40 × 10^3^ to 58,769.30 × 10^3^ g/mol ( × 1.5) for 65 and 95 °C, respectively]. These changes in molar masses were accompanied by an increase in the polydispersity index (PI) of these polymers (from 4.68 to 6.32 for 65 and 95 °C, respectively), reflecting an increase in their heterogeneity (Table 4).

**Table 4:** Macromolecular features of polymeric proteins in heat-treated DMLP.

Heat treatment	M_w_(a) ( × 10^4^ g/mol)	M_n_(a) ( × 10^3^ g/mol)	R_Gw_(a) (nm)	R_Gn_(a) (nm)	PI(b)	*v*(c)
Control	NE(d)	NE	NE	NE	NE	NE
65 °C/15min	18695.57 ± 0.69^c^	39914.40 ± 1.56^d^	134.6 ± 2.8^c^	82.5 ± 4.3^b^	4.68 ± 0.25^c^	0.347
75 °C/15min	20064.57 ± 1.75^c^	44144.70 ± 2.16^c^	126.1 ± 5.8^d^	83.2 ± 5.7^b^	4.55 ± 0.45^c^	0.333
85 °C/15min	27205.92 ± 1.93^b^	48075.00 ± 1.73^b^	148.4 ± 3.9^b^	80.5 ± 3.9^c^	5.66 ± 0.45^b^	0.315
95 °C/15min	37113.36 ± 4.82^a^	58769.30 ± 2.41^a^	171.3 ± 4.6^a^	85.9 ± 4.0^a^	6.32 ± 0.86^a^	0.314

Different letters within a column indicate significantly different values (p < 0.05).

(a) Mean ± standard deviation (n = 3) of M_w_ (weight-average molar mass), M_n_ (number-average molar mass), R_Gw_ (weight-average radius of gyration) and R_Gn_ (number-average radius of gyration).

(b) Polydispersity index: M_w_/M_n_.

(c) Conformation plot slope (molecular shape).

(d) Non-Estimable (too very low concentration signal).

At the same time, the distribution of the radii of the gyration of polymeric proteins also increased under the effect of heat treatment of insect meals. The weight-average radius of gyration (R_Gw_) thus increased from 134.6 to 171.3 nm for temperatures increasing from 65 to 95 °C. The same phenomenon was observable from the values of R_Gn_ [from 82.5 to 85.9 nm for 65 and 95 °C, respectively].

From these molar mass and size distribution data, we estimated the shape of these protein polymers by measuring the dependence of their size on their molar masses. Indeed, it was possible to fit the linear relationship of the logarithmic form as follows:

(Eq. 5) *R*_*G*_ = *KM*^*v*^

For macromolecular assemblies, the log–log plot of R_G_ *vs.* M allowed estimation of the shape of these polymers. Increasing values of the slope *v* progress from spherical (*v* < 0.35) to random coil (0.40 < *v* < 0.50) to linear conformations (*v* > 0.60–1.0) of polymers and aggregates.

These values, all less than 0.350, indicate that in all samples, the molecular dimensions of the proteins increase only slowly with their molecular mass and that consequently, these molecules have a compact structure. The increase in temperatures applied to insect larvae caused a decrease in this hydrodynamic parameter *v* (Table 4). Thus, *v* decreased from 0.347 to 0.314 for temperatures increasing from 65 to 95 °C, suggesting a significant increase in polymer compactness.

#### 3.3.3 Oxidation of DMLP induced by heat treatment

The polymeric proteins observed after the heat treatment of insect larvae disappeared if a chemical reduction step of the extract was carried out using DTT (Table 4). Thus, for all characterised samples, the P/M ratio decreased significantly after the DTT reduction step. For example, it went from 0.53 to 0.04, from 0.38 to 0.03 and from 0.12 to 0.02 for the treatments carried out at 95, 85 and 75 °C, respectively. This almost complete disappearance of protein polymers following DTT treatment resulted in an almost complete disappearance of the light scattering signal (Fig. 10f–h,j red lines *vs.* black lines) and, at the same time, in an increase in the quantity of monomers detected by UV (Fig. 10e–g,i red lines *vs.* black lines). These observations suggest that the protein polymers formed following heat treatments result from the formation of intermolecular protein disulphides.

**Fig. 11:**
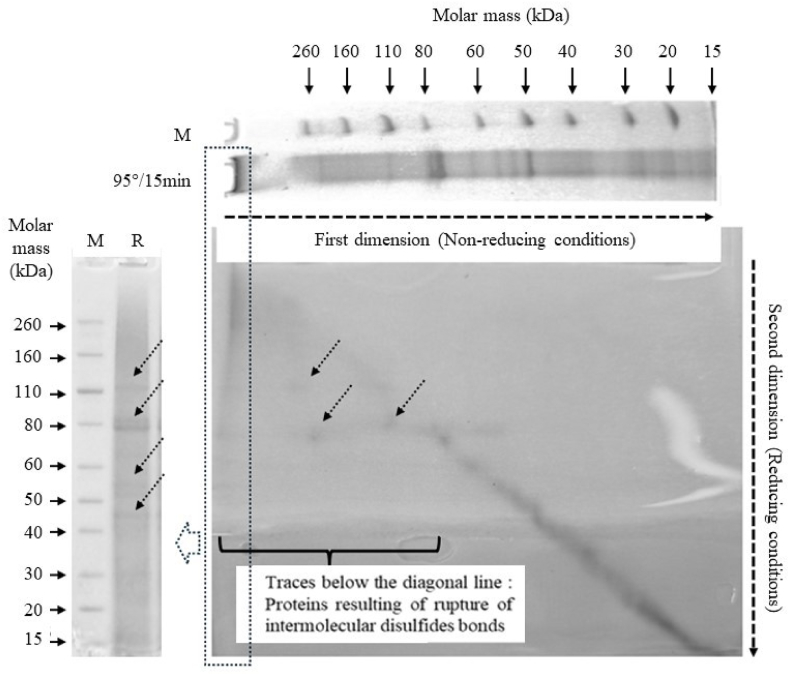
Diagonal gel electrophoresis of DMLP heat treated at 95 °C for 15 min. Protein markers (M line) with corresponding molar masses in kDa (black arrows). Major proteins resulting from the cleavage of intermolecular disulfide bonds (dotted arrows). Track R shown on the left of the figure corresponds to the separation under reduced conditions (+DTT) of the polymeric proteins initially present in the well of the first dimension (non-reduced conditions). This track corresponds in more detail to the dotted box of the diagonal gel.

This conclusion was confirmed by the implementation of diagonal gel electrophoresis. Diagonal electrophoresis is a sequential non-reducing analysis followed by a reducing gel analysis procedure that enables the identification of proteins that form inter-protein disulphides. When the gel is stained for total protein, the dominant feature is a diagonal line caused by most proteins running at the same molecular weight during both runs. Those proteins with inter-disulphides migrate faster in the second reducing separation, as the disulphide is chemically reduced, meaning they run at a lighter mass and thus off of the diagonal. As shown in Fig. 11, several proteins were detected outside the diagonal, resulting from chemical reduction by DTT of oligomeric and polymeric forms

The formation of intermolecular S-S bonds in proteins from heat-treated meals was responsible for the appearance of polymers whose cross-linking (formation of a covalent network) resulted in an increase in the compactness of these protein particles, as we already observed by measuring the hydrodynamic parameter *v* (Table 3).

**Fig. 12:**
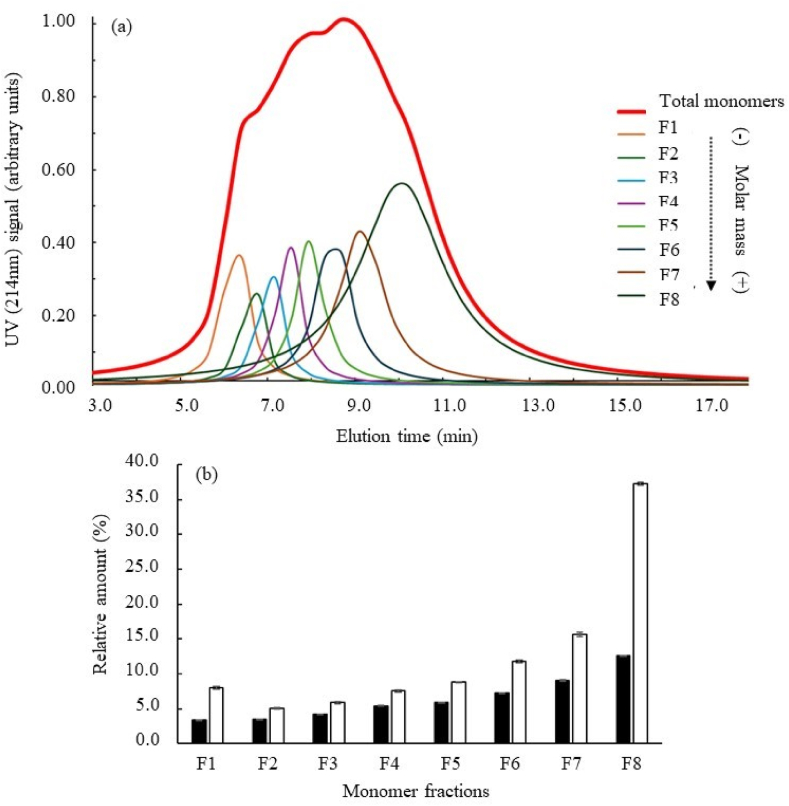
(a) Characterisation and (b) quantification of the different monomeric protein fractions (from F1 to F8) following the deconvolution of the concentration signal (UV signal) of the fractogram obtained from DMLP heat treated at 95 °C for 15 min (Black bars) Relative amounts before chemical reduction (DTT) of polymers, and (White bars) relative amounts after chemical reduction (DTT) of polymers.

The chemical reduction of protein polymers formed following heat treatment caused the reappearance of the monomers constituting these molecular structures, which was quantitatively characterised by comparison of the deconvoluted UV signals (Fig. 12a). Thus, several monomeric protein fractions of increasing molecular mass (from F1 to F8) were quantified (relative amounts) before and after the chemical reduction of the polymers (Fig. 12b).

The results obtained from the treatment at 95 °C for 15 min (heat treatment allowing the production of significant quantities of polymers) demonstrated that the chemical reduction of polymeric proteins generated a non-homogeneous release of monomers (Fig. 12b). Thus, following the rupture of the intermolecular S-S bonds by DTT, the monomeric fractions of high molar masses (especially F8) were over-represented in the extract. Thus, fraction 8, which represented ≈13 % of the monomers present before the chemical reduction, represented more than >37 % of the monomers quantified after the depolymerisation of the polymeric fraction (i.e. an enrichment of more than 24 % following the chemical reduction step) (Fig. 12b). These results are consistent with previous observations made during SDS-PAGE separation (Fig. 7). Thus, based on the staining intensity, the vast majority of proteins observed after separation under reducing conditions had high molar masses (≥80 kDa) (Fig. 11 track R).

These structural modifications of the DMLPs described here are reminiscent of those that we have already been able to highlight in other protein models present in particular in plants. Thus, if we observe, for example, the reserve proteins (gliadins and glutenins) of wheat grains, we can highlight a phenomenon of “hyperaggregation” of these important proteins during the last phase of grain maturation (desiccation phase) (Aussenac et al., 2018). As we have been able to demonstrate, during this specific phase, in which the wheat grain naturally dehydrates (i.e., the moisture content drops from 40 % to 12–13 %) under the influence of environmental parameters, the different protein subunits of gliadins and glutenins aggregate (by strengthening non-covalent interactions) and polymerize (formation of new covalent interactions) to form a three-dimensional protein network. Within the polymeric prolamins (glutenins), the aggregation of these proteins results from the strengthening of hydrogen bonds, electrostatic interactions, and hydrophobic bonds from the repeatable domains of the constituent subunits under the effect of the massive water loss. This aggregation leads in a second step to the formation of new intermolecular disulfide bonds stabilizing the structures also called glutenin macropolymers (GMP) in the form of condensed particles (*v* < 0.33). Beyond the fact that this “hyper-aggregation” phenomenon profoundly modifies the solubility of the storage proteins (significant decrease in the solubility of all prolamins), it is responsible for the acquisition of the majority of the techno functional properties of the prolamins thus organized (Aussenac et al., 2020). Even if the technological consequences appear opposite for DMLPs and wheat prolamins (i.e. loss of functionalities for the former and increase in rheological performances for the latter), the underlying biochemical mechanisms of aggregation and polymerisation are very close. In order to increase our knowledge of the phenomena characterized during our work, it would be important to identify, as in the case of wheat prolamins (GMP) 5, the protein subunits involved in these aggregation and polymerisation phenomena (M > 80 kDa) as well as the specific position of the free -SH groups responsible for the formation of new intermolecular disulfide bridges.

## 4 Conclusion

In this study, we investigated the effects of heat treatment on the aggregation (protein–protein interactions through non-covalent bonds) and polymerisation (protein–protein interactions through covalent bonds) behaviours of mealworm protein extracts. Heat treatment significantly affected intrinsic fluorescence and surface hydrophobicity; most of the hydrophobic residues were exposed upon unfolding, leading to reduced solubility because the nonpolar groups formed intermolecular hydrophobic bonds and further increased protein aggregation. Moreover, our results demonstrated that proteins with cysteine groups underwent polymerisation under heat, causing a thiol-disulphide exchange reaction and forming a strong polymer structure. Upon denaturation, proteins unfolded and revealed the thiol groups available for intermolecular interactions. The establishment of these covalent intermolecular interactions during heat treatment preferentially mobilised protein subunits with molar masses ≥80 kDa. The development of an original analytical approach (AF4-MALLS-UV) enabled us to overcome the limits of the characterisation of protein assemblies present in insect larvae extracts. Indeed, this methodology (i) quantified and characterised the aggregated protein forms dissociable after denaturation (i.e. after the action of urea), determining the P/M ratio and, at the same time, (ii) quantified and characterised the polymerised protein forms dissociable after chemical reduction of intermolecular bonds (i.e. after the action of DTT), determining the molecular mass and size distribution (i.e. MMD and DRG) and conformation of different elements. Major insect larvae protein functionalities were closely related to these chemical properties. Given its capabilities and performance, this new analytical approach should make it possible to quantify and characterise the effects of the different processing methods (such as defatting techniques and thermal and other non-thermal treatments for protein extraction) used for the preparation of protein extracts from insect larvae and, more broadly, to optimise the functionalities of these proteins by limiting the undesirable effects of these processing methods.

## CRediT authorship contribution statement

**Ariel Anouma:** Investigation, Writing – original draft, preparation. **Céline Niquet-Léridon:** Supervision, Writing – review & editing. **Bénédicte Lorrette:** Project administration, Writing – review & editing. **Thierry Aussenac:** Conceptualization, Supervision, Methodology, Writing – review & editing.

## Funding sources

This work was supported by the 10.13039/501100003032National Association for Research and Technology Convention CIFRE N° 2023/0059.

## Declaration of competing interest

The authors declare the following financial interests/personal relationships which may be considered as potential competing interests: Ariel ANOUMA reports financial support was provided by National Association of Technical Research. If there are other authors, they declare that they have no known competing financial interests or personal relationships that could have appeared to influence the work reported in this paper.

## Data Availability

Data will be made available on request.
